# Reconstruction of Abdominal Wall Defects Using a Pedicled Anterolateral Thigh Flap including the Vastus Lateralis Muscle: A Report of Two Cases

**DOI:** 10.1155/2016/8753479

**Published:** 2016-12-15

**Authors:** Kiyoko Fukui, Masaki Fujioka, Satoko Ishiyama

**Affiliations:** ^1^Department of Plastic and Reconstructive Surgery, National Hospital Organization Nagasaki Medical Center, Nagasaki, Japan; ^2^Department of Plastic and Reconstructive Surgery, Nagasaki University, Nagasaki, Japan; ^3^Department of Plastic and Reconstructive Surgery, Clinical Research Center, National Hospital Organization Nagasaki Medical Center, Nagasaki, Japan

## Abstract

The purpose of abdominal wall reconstruction is to prevent hernias and protect the abdominal viscera. In cases involving full-thickness defects of the rectus abdominis muscle, the muscle layer should be repaired. We present 2 cases in which full-thickness lower rectus abdominis muscle defects were reconstructed using vastus lateralis-anterolateral thigh flaps. The pedicled vastus lateralis-anterolateral thigh flap provides skin, fascia, and muscle tissue. Furthermore, it has a long neurovascular pedicle and can reach up to the periumbilical area and cover large defects. We consider that this muscle flap is a good option for repairing full-thickness lower abdominal defects.

## 1. Introduction

The surgical excision of abdominal tumors, such as desmoid tumors, cancer, and metastatic disease, can result in full-thickness abdominal wall defects, which require repairing.

Abdominal wall reconstruction aims to restore musculofascial integrity, prevent hernias, and protect the abdominal viscera.

A variety of abdominal wall reconstruction techniques have been reported [1–3]; however, recent articles have indicated that reconstruction procedures involving muscle flaps help to prevent hernias in the long term [2]. In cases involving full-thickness rectus abdominis muscle defects, muscle layer reconstruction should be performed with muscle flaps.

We present 2 cases in which full-thickness lower rectus abdominis muscle defects were reconstructed using vastus lateralis-anterolateral thigh flaps.

## 2. Case Presentation

### 2.1. Case 1

A 19-year-old female visited the Department of General Surgery due to a nodule in her lower left abdomen. A histological analysis of the biopsy specimen suggested that the nodule was a desmoid tumor. She was referred to the Department of Plastic and Reconstructive Surgery to have the tumor removed.

During the first examination, a hard mass that exhibited poor mobility was detected in the lower left abdomen.

Magnetic resonance imaging (MRI) revealed that the tumor was 7 × 7 cm in size, displayed isodensity compared with muscle, and occupied the full thickness of the rectus abdominis muscle.

**Figure 1:**
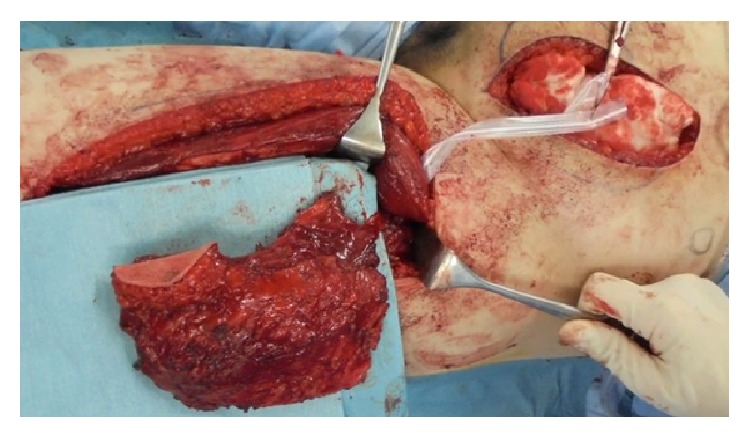
A pedicled vastus lateralis-anterolateral thigh flap was elevated from the left thigh. The flap was harvested together with a motor nerve.

**Figure 2:**
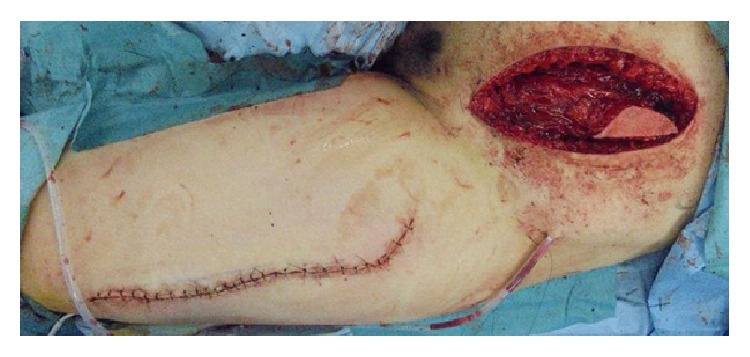
The flap was tunneled under the rectus femoris muscle and transferred to the abdominal wall defect.

Wide surgical resection was performed with a 3 cm superior margin. The inferior surgical margin was located at the pubic symphysis, and the resected area included the anterior and posterior rectus sheath, peritoneum, and the full thickness and width of the left rectus abdominis muscle. The peritoneum was closed primarily, and a muscle defect measuring 9 × 13 cm was repaired with an 11 × 15 cm pedicled anterolateral thigh flap, which included the vastus lateralis muscle (Figure 1). The descending branch of the deep femoral artery was dissected up to its origin, and the flap was harvested together with a motor nerve from the left thigh. The flap was tunneled under the rectus femoris muscle to medialize the neurovascular pedicle and then transferred to the abdominal wall defect (Figure 2). The vastus lateralis muscle was sutured to the remaining rectus abdominis muscle.

**Figure 3:**
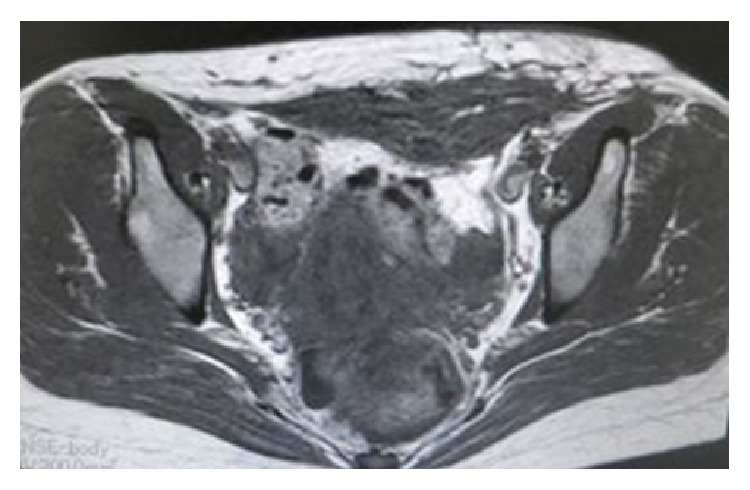
An MRI scan at one postoperative month showed that the rectus abdominis muscle defect had been reconstructed with the vastus lateralis muscle flap.

**Figure 4:**
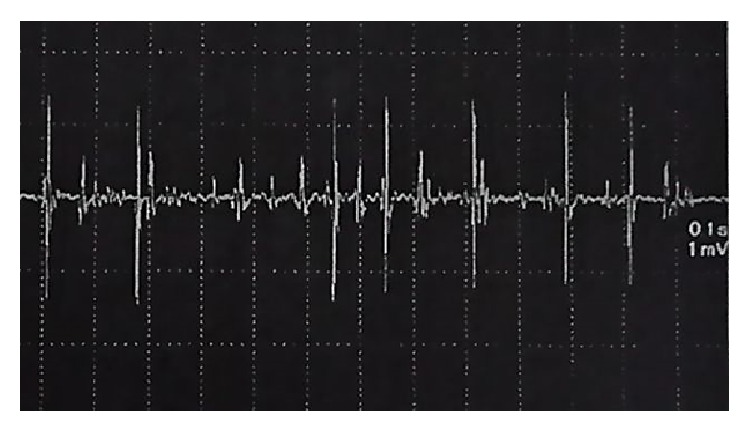
The transferred vastus lateralis muscle underwent voluntary contraction according to electromyography at 6 postoperative months.

The patient was able to walk 7 days after surgery and was discharged 2 weeks later. An MRI scan obtained at one postoperative month showed that the rectus abdominis muscle defect had been reconstructed with the vastus lateralis muscle flap, and no hernias had developed (Figure 3). When the patient flexed the hip joint and kept her abdominal muscles contracted, the transferred vastus lateralis muscle underwent voluntary contraction according to electromyography performed at 6 postoperative months (Figure 4).

### 2.2. Case 2

A 53-year-old male developed recurrent bladder cancer. He had undergone surgery to remove his bladder and create an ileal orthotopic neobladder 3 years ago. A computed tomography scan showed thickening in the anterior part of the neobladder and invasion into the rectus abdominis muscle (dimensions of the affected area: 58 × 30 mm). He was referred to the Department of Plastic and Reconstructive Surgery to undergo abdominal wall reconstruction.

**Figure 5:**
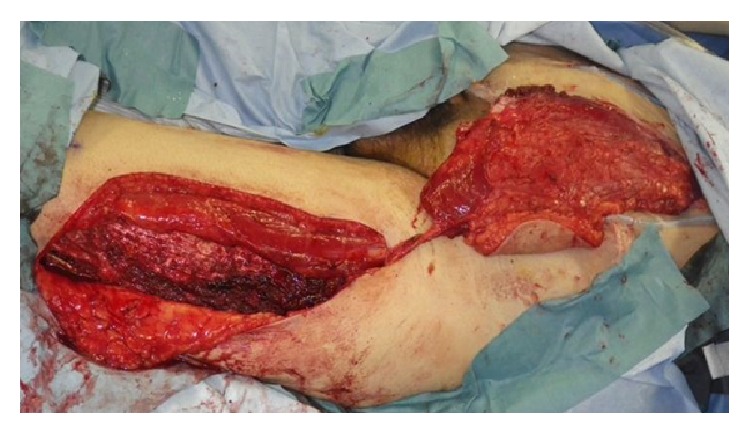
A pedicled vastus lateralis-anterolateral thigh flap was elevated with a motor nerve.

Wide tumor resection was performed (the resected area included part of the anterior neobladder, peritoneum, and the full thickness of both rectus abdominis muscles). After closing the anterior neobladder defect, a 7 × 15 cm defect in the peritoneum and muscle remained. The patient did not have any skin defects. A pedicled vastus lateralis-anterolateral thigh flap (size of the muscle and fascia component: 10 cm × 20 cm) was harvested from the left thigh (Figure 5). The flap was transferred to the abdominal defect through a subcutaneous tunnel. The fascia lata and vastus lateralis muscle were sutured to the remaining anterior rectus sheath and abdominal muscle.

One month later, the patient had not suffered any hernias and was able to walk without any problems. An MRI scan obtained at one month after surgery did not show any hernias and demonstrated that the patient's abdominal wall defect had been successfully reconstructed with the muscle flap.

## 3. Discussion

During abdominal wall reconstruction, it is essential to restore the structural integrity of the abdominal wall to prevent hernias and protect the abdominal viscera [1–3]. Full-thickness abdominal wall defects should be reconstructed by repairing the skin, soft tissue, and supportive tissue (the fascia, muscle, and peritoneum). Numerous flaps that can be used to repair such defects have been reported, but recently it has been suggested that reconstruction of the muscle layer is indispensable for preventing hernias in the long term [2–4]. The “like-with-like” principle of reconstructive surgery should be adhered to by reconstructing all components of abdominal defects, and muscle layer reconstruction is especially important in cases of full-thickness rectus abdominis muscle defects [2–4].

Various thigh muscle flaps that can be used to reconstruct the lower abdomen have been described, including tensor fascia lata (TFL), gracilis, rectus femoris, and vastus lateralis muscle flaps [1–3]. TFL muscle flaps have a large skin paddle but are associated with distal skin necrosis. Gracilis muscle flaps are limited in size and their arc of rotation and provide unreliable blood flow to the distal skin island. Rectus femoris muscle flaps have a large arc of rotation but are associated with severe donor site morbidity [2, 3].

We used a pedicled vastus lateralis-anterolateral thigh flap to reconstruct the abdominal wall. Such flaps can be used to repair large muscle layer defects as they contain sufficient muscle volume, whereas gracilis and TFL muscle flaps have relatively little muscle volume.

Pedicled vastus lateralis-anterolateral thigh flaps can provide skin, fascia, and muscle tissue. Furthermore, these flaps have a long neurovascular pedicle, reach up to the periumbilical area, and can cover large defects [4, 5]. The main disadvantages of such flaps are that their perforators can vary quite markedly, and they are associated with donor site morbidity and can bulge at rest [4].

Vranckx et al. described the successful use of a pedicled innervated vastus lateralis muscle flap and a fasciocutaneous anterolateral thigh flap during dynamic reconstruction [4]. In our case, the transferred vastus lateralis muscle underwent voluntary contraction (according to electromyography) when the patient flexed the hip joint and kept her abdominal muscles contracted.

Free tissue transfer can also be used for abdominal reconstruction [2, 3]. When free latissimus dorsi muscle flaps or vastus lateralis muscle flaps are transferred, the attached motor nerves can be connected with intercostal nerves. However, even when the period from the cutting of the transferred nerve to its reinnervation is short, motor nerve transection can lead to muscle denervation and atrophy. We harvested a pedicled vastus lateralis-anterolateral thigh flap with an intact motor nerve, and this technique can help to avoid muscle atrophy.

In our cases, no donor site morbidity was observed. Harvesting the vastus lateralis muscle is reported to result in limited donor site functional morbidity [2, 4].

A pedicled anterolateral thigh flap containing the vastus lateralis muscle is a good option for reconstructing complex lower abdominal wall defects.

## 4. Conclusion

We used a pedicled vastus lateralis-anterolateral thigh flap for muscle layer reconstruction. Pedicled vastus lateralis-anterolateral thigh flaps can provide skin, fascia, and muscle tissue. In addition, they have a long neurovascular pedicle, reach up to the periumbilical area, and can be used to cover large defects. We consider that such flaps are useful for repairing lower abdominal full-thickness defects.
